# Prognostic impact and implications of extracapsular lymph node spread in Borrmann type IV gastric cancer

**DOI:** 10.18632/oncotarget.18400

**Published:** 2017-06-07

**Authors:** Rui-Zeng Dong, Jian-Min Guo, Ze-Wei Zhang, Yi-Min Zhou, Ying Su

**Affiliations:** ^1^ Department of Abdominal Surgery, Zhejiang Cancer Hospital, Hangzhou, People’s Republic of China; ^2^ Department of Pathology, Zhejiang Cancer Hospital, Hangzhou, People’s Republic of China

**Keywords:** Borrmann type IV gastric cancer, extracapsular lymph node spread, curative resection, prognosis, metastasis

## Abstract

The purpose of this study was to evaluate the relationship between extracapsular lymph node spread (ECS) and clinicopathology and its influence on the prognosis in patients with Borrmann type IV gastric cancer. Between 2002 and 2014, clinical data were reviewed from 486 patients with Borrmann type IV gastric cancer who underwent curative resection. Of the 486 patients, lymph node metastasis was found in 456. ECS was detected in 213 (46.7%) patients with lymph node metastasis. A positive lymph node with ECS was significantly correlated with the N category, lymphatic/venous invasion, tumor location, and TNM stage. For the whole patients, the mean OS was 34.7 months, and the 5-year OS rate was 15.5%. The 5-year OS rate of node-negative patients was 48%, for node-positive patients without ECS 18.7%, and for node-positive patients with ECS 5.7% (*P* = 0.000). In a multivariate analysis, adjusted for tumor location, lymphatic/venous invasion, body mass index (BMI), and TNM stages, ECS remained an independent prognostic factor. For patients with the same N category and TNM stage, those with ECS still had a worse survival rate. Recurrent sites were confirmed in 367 patients. The most frequent recurrent site was the peritoneum. There was a significant difference between ECS+ (*N* = 150) and ECS- (*N* = 142) patients (*P* = 0.008). Our results suggested that ECS was an independent prognostic value for Borrmann type IV gastric cancer patients with curative resection and a subgroup indicated a significantly worse long-term survival for patients with the same N or TNM stages. ECS+ was an adverse factor for peritoneal metastasis.

## INTRODUCTION

The incidence of gastric cancer has decreased over the past decades, but it is still the second leading cause of cancer-related death in China [1]. Borrmann type IV gastric cancer accounts for about 8–13% of all gastric cancers [2, 3]. Surgery is the primary treatment method to cure this type of gastric cancer, and complete resection is considered as a standard goal. The prognosis remains poor due to early detection delay and aggressive tumor behavior. Most of the patients develop peritoneal and/or lymph node metastases after surgery. The 5-year OS ranges from 8% to 27.6% [3, 4]. Lymph node metastasis has been identified as an independent prognostic indicator in patients with Borrmann type IV gastric cancer [5]. However, in the current gastric cancer stage system, we focus only on the number of metastatic lymph nodes, and the characteristics of metastatic lymph nodes are not considered. The impact of extracapsular lymph node spread (ECS) has been studied in several cancers, including colorectal, head/neck, and bladder cancer [6–8]. Several studies have found a significant correlation of ECS with survival in gastric cancer. However, in Borrmann type IV gastric cancer, the prevalence and significance of ECS have not been well established. Therefore, in the present study, we explored the prevalence of ECS and its correlations with clinicopathological characteristics to determine the predictive value for survival and peritoneal recurrence in Borrmann type IV gastric cancer.

## RESULTS

### Characteristics of patients and resected lymph nodes

Of the 485 patients with Borrmann type IV gastric cancer who underwent curative gastrectomy, there were 328 men (67.6%) and 157 women (32.4%) with a mean age of 57.3 years (range 19–79, SD ± 11.9).

Total gastrectomy was performed in 339 patients (69.9%) and subtotal gastrectomy was performed in 146 patients (30.1%). Combined multiple organ resection was carried out in 83 patients (17.1%), including splenectomy (40 patients), distal pancreatectomy with the spleen (25 patients), colectomy (10 patients), pancreaticoduodenectomy (5 patients), and partial hepatectomy (3 patients). All these operations were accompanied by a D2/D2+ lymphadenectomy. The mean number of resected lymph nodes per patient was 33.5 (range 6–82, SD ± 13.1). Pathologic lymph node metastasis was detected in 456 patients (94.0%). The mean number of metastatic lymph nodes for these patients was 15.4 (range 1–79, SD ± 12.5). According to the AJCC staging system, the numbers of lymph node metastasis in 0, 1, 2, and 3 were 29 (5.9%), 39 (8%), 92 (19%), and 325 (67.1%).

### Clinicopathological features between ECS-positive and ECS-negative patients

In the 456 patients with lymph node metastasis, ECS was detected in 213 patients (46.7%). Clinicopathological characteristics are presented in Table 1. Factors related to ECS were N category, lymphatic or venous invasion, tumor location, and TNM stage.

**Table 1:** Clinicopathological features between extracapsular lymph node spread in the positive and negative groups of 456 patients with lymph node metastasis

Categories	Extracapsular lymph node spread			
	Negative (*n* = 243) No. (%)	Positive (n = 213) No. (%)	χ^2^	*P* value
Sex				
Male	163 (53.1%)	144 (46.9%)		
Female	80 (53.7%)	69 (46.3%)	0.014	0.905
Age				
≥ 60	120 (53.3%)	105 (46.7%)		
*<* 60	123 (53.2%)	108 (46.8%)	0.000	0.985
Histological type				
Papillary or tubular	196 (55.7%)	156 (44.3%)		
Ring cell	34 (48.6%)	36 (51.4%)		
Mucinous	12 (37.5%)	20 (62.5%)		
Others	1 (50.0%)	1 (50.0%)	4.649	0.199
Differentiation				
Well	1 (25%)	3 (75%)		
Moderate	13 (59.1%)	9 (40.9%)		
Poor	299 (53.3%)	201 (46.1)	1.584	0.453
pT category				
T2-3	10 (62.5%)	6 (37.5%)		
T4a	205 (53.9%)	175 (46.1%)		
T4b	28 (46.7%)	32 (53.3%)	1.669	0.434
pN category				
N1	36 (92.3%)	3 (7.7%)		
N2	63 (68.5%)	29 (31.5%)		
N3	144 (44.3%)	181 (55.7%)	42.913	0.000
Lymphatic or venous invasion				
Negative	121 (62.7%)	72 (37.3%)		
Positive	122 (46.4%)	141 (53.6%)	11.891	0.001
Neural invasion				
Negative	68 (56.2%)	53 (43.8%)		
Positive	175 (52.2%)	160 (47.8%)	0.560	0.454
CEA				
Normal	195 (54.5%)	163 (45.5%)		
Abnormal	48 (49%)	50 (51%)	0.931	0.334
CA 19-9				
Normal	198 (53.2%)	174 (46.8%)		
Abnormal	45 (53.6%)	39 (46.4%)	0.003	0.954
BMI				
≥ 18.5	213 (54.2%)	180 (45.8%)		
*<* 18.5	30 (47.6%)	33 (52.4%)	0.944	0.331
Location				
Upper	20 (64.5%)	11 (35.5%)		
Middle	21 (67.7%)	10 (32.3%)		
Lower	41 (65.1%)	22 (34.9%)		
Upper-middle	47 (47.5%)	52 (52.5%)		
Middle-lower	66 (57.4%)	49 (42.6%)		
UML	42 (38.9%)	66 (61.1%)		
Remnant	6 (66.7%)	3 (33.3%)	19.456	0.003
TNM stage				
IIb	6 (75.0%)	2 (25.0%)		
IIIa	35 (85.4%)	6 (14.6%)		
IIIb	54 (69.2%)	24 (30.8%)		
IIIc	148 (45.0%)	181 (55.0%)	35.541	0.000

Extracapsular lymph node spread did not correlate with age, sex, histological type, tumor differentiation, T category, level of serum CEA and CA 19-9, and nutritional condition (BMI).

### Prognostic significance of ECS

**Figure 1:**
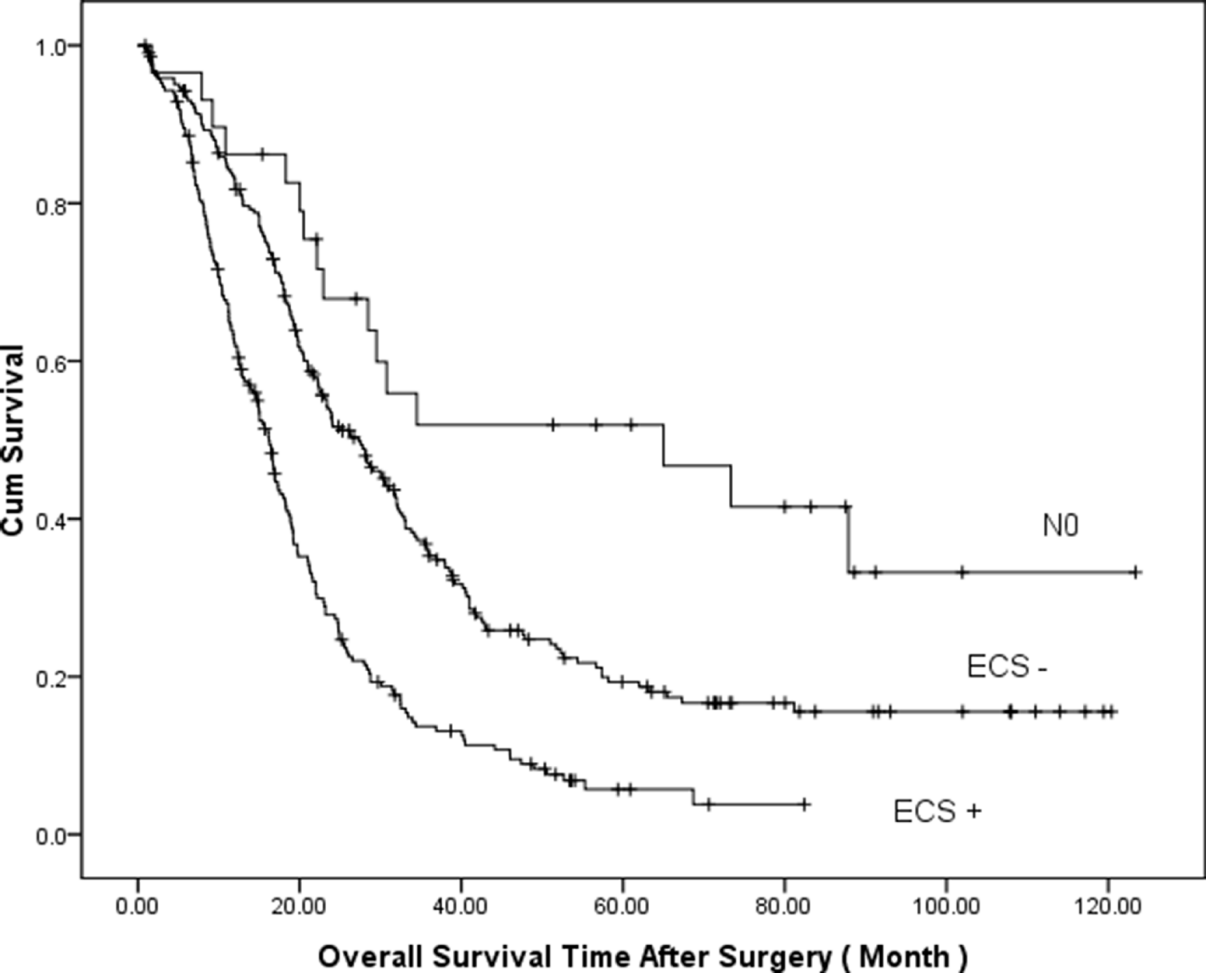
Comparison of cumulative survival based on lymph node status (*P* = 0.000, log-rank test)

The 5-year survival for the 486 patients with Borrmann type IV gastric cancer was 15.5%, with a mean survival time of 34.7 months (SD ± 1.79). In a univariate analysis (Table 2), T category, N category, lymphatic/venous invasion, neural invasion, CEA, CA 19-9, location, TNM stage, and ECS were associated with OS. Patients with extracapsular lymph node spread had a significantly worse prognosis than those without ECS. Survival time analysis based on lymph node status is shown in Figure 1: the 5-year survival rate for patients with ECS was 5.7% and the mean survival time was 21.0 months (SD ± 1.34). Patients with lymph node metastasis without ECS had a 5-year survival rate of 18.7% and the mean survival time was 40.6 months (± 2.62). Patients without lymph node metastasis had a 5-year survival rate of 48% and the mean survival time was 65.2 months (± 9.37). In a multivariate analysis adjusted for lymphatic/venous invasion, BMI, location, and TNM stage, the presence of ECS was an independent prognostic factor (Table 2).

**Table 2:** Univariate analysis and multivariate Cox analysis for prognostic factors

Categories	Univariate			Multivariate Cox analysis		
	Mean survival time ( month )	χ2	*P* value	HR	95% CI	*P* value
Sex						
Male	30.96 ± 1.96					
Female	34.11 ± 3.01	0.982	0.322	-	-	-
Age						
≥ 60	34.76 ± 2.57					
*<* 60	29.71 ± 2.19	2.028	0.154	-	-	-
Histological type						
Papillary/tubular	29.67 ± 1.79					
Ring cell	38.77 ± 5.51					
Mucinous	37.84 ± 4.75					
Others	29.53 ± 0.63	6.653	0.084	-	-	-
Differentiation						
Well/Moderate	39.35 ± 7.59					
Poor	31.88 ± 1.75	1.073	0.300	-	-	-
pT category						
T2-3	43.58 ± 7.35					
T4a	34.69 ± 1.97					
T4b	14.38 ± 1.32	49.369	0.000	-	-	-
pN category						
N1	52.74 ± 6.24					
N2	41.69 ± 4.18					
vN3	26.80 ± 1.79	32.767	0.000	-	-	-
Lymphatic/venous invasion						
Negative	39.67 ± 2.90					
Positive	27.05 ± 1.99	18.917	0.000	1.298	1.040-1.621	0.021
Neural invasion						
Negative	40.91 ± 3.98					
Positive	28.40 ± 1.64	6.807	0.009	1.166	0.910-1.496	0.225
CEA						
Normal	33.89 ± 1.95					
Abnormal	26.46 ± 3.38	7.129	0.008	1.120	0.865-1.450	0.389
CA 19-9						
Normal	34.73 ± 1.99					
Abnormal	20.86 ± 1.95	12.132	0.000	1.285	0.987-1.672	0.063
BMI						
≥ 18.5	33.79 ± 1.91					
*<* 18.5	22.98 ± 2.59	4.324	0.038	1.405	1.053-1.875	0.021
Location						
Upper	28.40 ± 3.89					
Middle	52.85 ± 8.13					
Lower	45.16 ± 5.24					
Upper-middle	30.84 ± 3.75					
Middle-lower	28.03 ± 1.88					
UML	18.48 ± 1.44					
Remnant	26.72 ± 7.38	43.995	0.000	1.140	1.062-1.224	0.000
TNM stage						
IIb	55.08 ± 10.55					
IIIa	52.02 ± 6.27					
IIIb	41.34 ± 4.50					
IIIc	26.37 ± 1.74	38.233	0.000	1.348	1.145-1.588	0.000
Extracapsular lymph node spread						
Negative	40.65 ± 2.62					
Positive	21.02 ± 1.34	42.246	0.000	1.588	1.277-1.973	0.000

**Figure 2:**
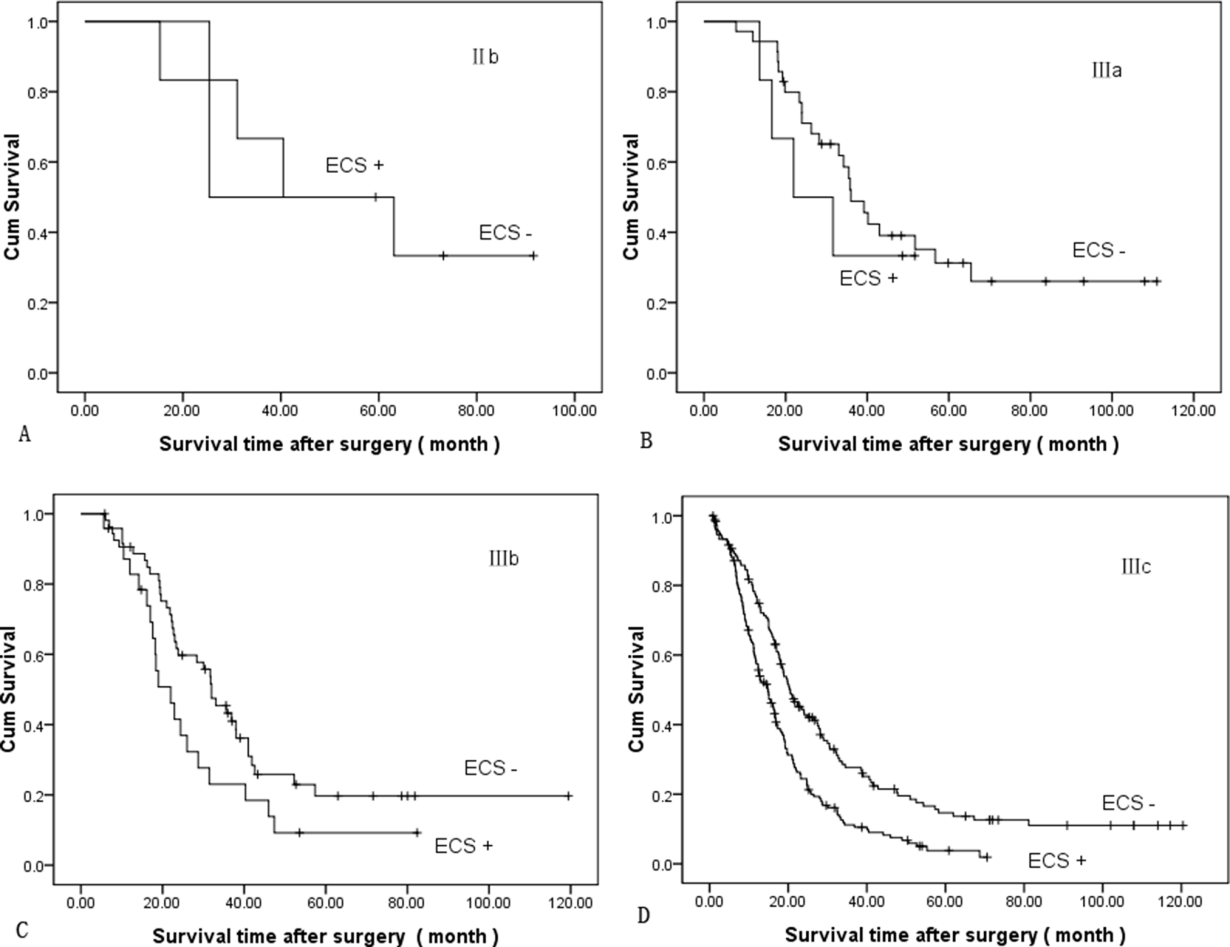
Comparison of cumulative survival between ECS+ and ECS- adjusted for TNM stage (*P* = 0.000, log-rank test): (**A**) stage II b, (**B**) stage III a, (**C**) stage III b (**D**) stage III c.

**Figure 3:**
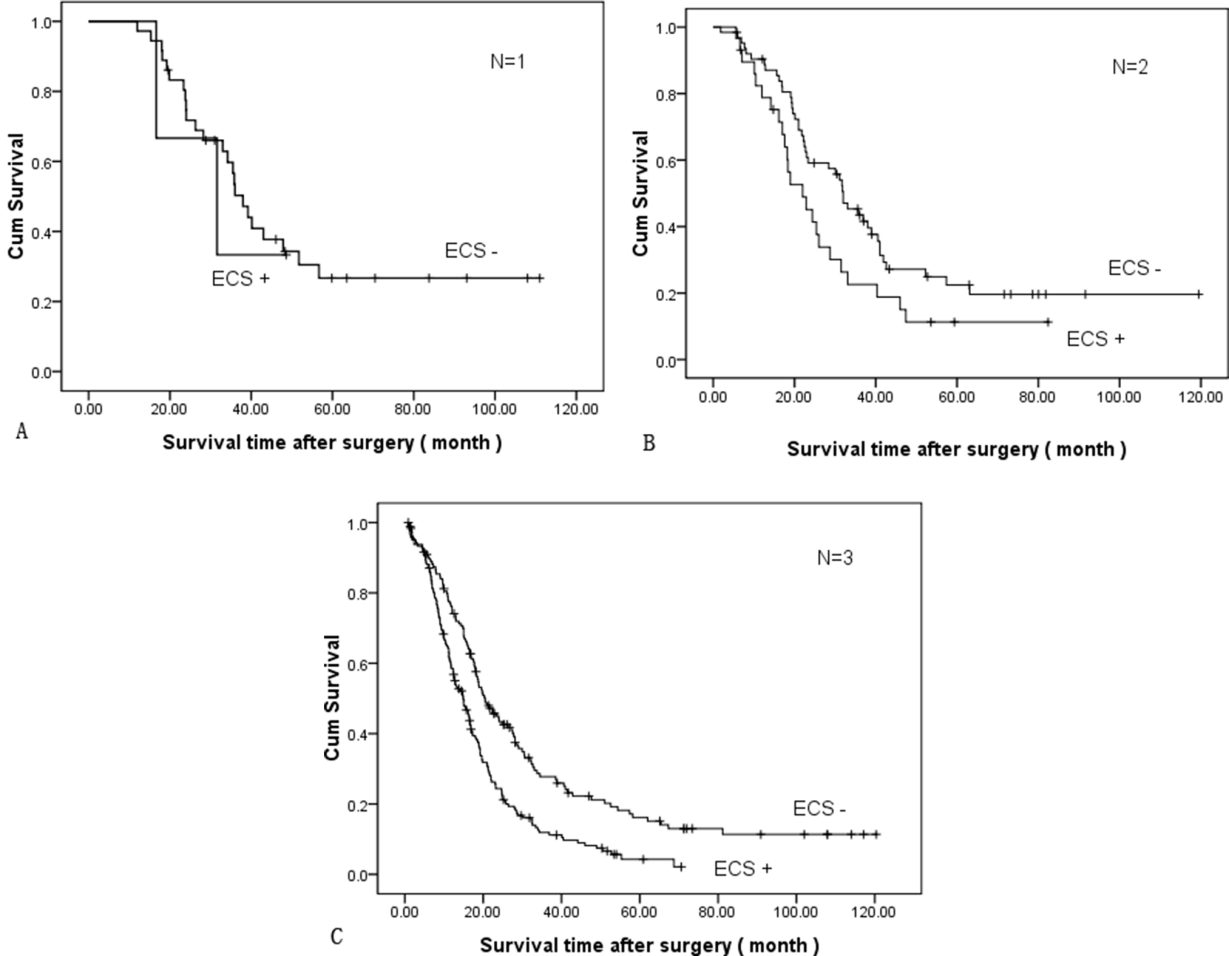
Comparison of cumulative survival between ECS+ and ECS- adjusted for N stage (*P* = 0.000, log-rank test): (**A**) *N* = 1, (**B**) *N* = 2, (**C**) *N* = 3.

Survival analyses were calculated among different subgroups, respectively. The cumulative survival between ECS+ and ECS- adjusted for the TNM stage showed ECS+ patients had a worse survival in the same TNM stage group (Figure 2). The mean survival time was 55.53 ± 11.9 months and 42.38 ± 12.0 months in stage IIb, 53.19 ± 6.66 months and 31.18 ± 6.33 months in stage IIIa, 45.98 ± 5.76 months and 28.11 ± 4.33 months in stage IIIb, and 34.0 ± 3.09 months and 18.79 ± 1.21 months in stage IIIc, respectively (*P* = 0.000, log-rank test). Cumulative survival between ECS+ and ECS- adjusted for the N stage showed ECS+ patients had a worse survival in the same N stage group (Figure 3). The mean survival time was 53.06 ± 6.44 months and 32.29 ± 7.54 months in stage N1, 45.99 ± 5.29 months and 28.71 ± 4.22 months in stage N2, and 34.57 ± 3.18 months and 19.12 ± 1.24 months in stage N3, respectively (*P* = 0.000, log-rank test).

Of the 456 patients with lymph node metastasis who underwent curative surgery, 367 patients’ recurrence was confirmed clinically or pathologically at the time of analysis. The most frequent recurrent site was the peritoneum (292, 79.6%). There was a significant difference between ECS+ (*N* = 150, 70.4%) and ECS- (*N* = 142, 58.4%) patients (χ^2^ = 7.081, *P* = 0.008), and the second most common was locoregional sites (151, 41%), including anastomotic recurrence and abdominal lymph node metastasis. Distant metastasis was found in 71 patients, and the most common sites were the liver, lungs, bones, and adrenal glands.

## DISCUSSION

TNM stage is still the critical prognosis factor for gastric cancer, but further research has reported that a variety of clinicopathologic factors were associated with prognosis [9–11]. These factors that impact the survival of patients with gastric cancer may be useful for individual treatment strategies. In head and neck cancer, ECS was considered an important adverse feature for postoperative systematic therapy/radiation therapy. However, in gastric cancer, no treatment guidelines have been established according to ECS. Lymphatic spread is the most common metastatic site and one of the most relevant prognostic factors for gastric cancer. The UICC and JCGC N staging system are based only on the number or the location of metastatic lymph nodes and do not consider the characteristics of the metastatic lymph node itself. Several studies have found a significant correlation with survival in gastric cancer, but it is not well known in Borrmann type IV gastric cancer. In our study, the patients with the same TNM stage or N stage had significantly different survival times between ECS+ and ECS- patients. The results showed that ECS can provide more detailed supplemental information for the TNM staging system and further evidence for individualized treatment.

The rate of lymph node metastasis was very high in Borrmann type IV gastric cancer because more patients were diagnosed in the advanced stage. In this study, pathologic lymph node metastasis was detected in 94.0% Borrmann type IV gastric cancer patients; this rate was similar to that reported by Yokota T [12]. Of the patients with lymph node metastasis, ECS+ was detected in 46.7% patients and the rate was significantly increased in the patients with N3, lymphatic/venous invasion or tumor infiltration the whole stomach. ECS is a common phenomenon in metastatic lymph nodes. Extracapsular lymph node spread is a significant risk factor for peritoneal metastasis of gastric cancer as reported by several authors [13, 14]. The rate of postoperative peritoneal recurrence is higher in Borrmann type IV gastric cancer than other Borrmann type gastric cancer. In Borrmann type IV gastric cancer patients, ECS+ patients had a greater risk of peritoneal recurrence than ECS- patients after curative resection. In our study, peritoneal recurrence occurred in 70.4% of ECS+ patients but only 58.4% of ECS- patients. The iatrogenic spread may be an important factor besides the aggressive biological behavior of the tumor. The result suggested that en bloc lymphadenectomy is more important for radical gastrectomy in Borrmann type IV gastric cancer. During the operation, it is best to avoid the exposure of the surgical instruments to the adipose tissue around the lymph nodes to prevent iatrogenic tumor dissemination. Intraoperative peritoneal lavage with distilled water or antitumor drugs and postoperative comprehensive treatment including chemotherapy and intraperitoneal perfusion may reduce the risk of recurrence in the abdominal cavity. Using anticancer drugs to remove the perinodal cancer cells before surgery might decrease the risk of cancer cell dissemination to improve long-term survival. However, we have no effective way to determine extracapsular lymph node spread before surgery. The use of S-1 alone as a neoadjuvant chemotherapy regimen was disappointing for Borrmann type IV gastric cancer in the JCOG 0002 trial [15]. Preoperative chemotherapy with S-1 + CDDP may be a promising regimen [16]. Further clinical research should be performed.

Treatment options for patients with Borrmann type IV gastric cancer are challenging and controversial because of the poor prognosis. It is promising that the survival outcome of Borrmann type IV gastric cancer potentially improved using multimodality treatment has been reported [17]. Early detection of Borrmann type IV gastric cancer is still difficult because the cancer cells dispersedly invade the mucosa propria without prominent ulceration or elevation on the mucosal surface in the early stage. Borrmann type IV gastric cancer shows more aggressive biological behavior and a worse prognosis than other Borrmann types of advanced gastric cancer. When diagnosed, cancer cells were often found to penetrate the serosa with lymph node metastasis. The OS of Borrmann type IV gastric cancer reported by different authors was heterogeneous. The curative resection rate is important, and the prognosis may be ameliorated with complete resection. The complete resection rate was 31% and the OS rate was 8% as reported by Schauer et al [4]. However, in a multivariate analysis, curability was not a significant predictor of survival [18]. Another reason could be attributed to the different constituent ratios of the tumor stages. When stratified by stage, OS time is similar in different reports. The 5-year survival rate was 49.8% for stage II, 36.4% for stage IIIa, and 15.2% for stage IIIb, respectively, as reported by An JY, et al [3]. In our study, the 5-year survival rate for the 486 patients with Borrmann type IV gastric cancer was 15.5% and patients with lymph node metastasis without extracapsular lymph node spread had a 5-year survival time of 18.7% while the 5-year survival time for patients with extracapsular lymph node spread was only 5.7%. However, in the patients without lymph node metastasis, the 5-year survival time reached 48%. From the results of survival, we can infer that most of the curative resections for advanced Borrmann type IV gastric cancer patients were actually non-curative resections, especially for ECS+ patients. A significant survival advantage for patients with Borrmann type IV gastric cancer can be achieved only after an actual complete resection, in other words, in the early stage. Kikuchi reported that the 5-year survival rate was 35.7% for the n0 patients, 27.8% for the n1 patients, 18.2% for the n2 patients, and 0% for the n3 or n4 patients [19]. The results of the present studies indicate that patients with Borrmann type IV gastric cancer may indeed be cured by curative surgery. It is encouraging that the prognosis for patients with Borrmann type IV gastric cancer was favorable if the tumor was removed before lymph node metastasis occurred. Therefore, early detection is important to improve the survival of patients. It is important to detect Borrmann type IV gastric cancer at an early stage and develop multimodality treatment strategies to achieve longer survival.

## MATERIALS AND METHODS

### Patients

Between January 2002 and December 2014, a consecutive series of 656 patients with Borrmann type IV gastric cancer underwent gastrectomy at Zhejiang Cancer Hospital. These patients constituted 8.2% of the 8036 with gastric cancer who underwent gastrectomy during the same period. The patients’ data were retrospectively reviewed. Among them, 485 patients were curative intent. These patients didn’t receive neoadjuvant chemotherapy and/or radiotherapy. Curative resection was defined as having no macroscopic residual tumor and free surgical margins at histology, along with the absence of distant metastasis, including negative peritoneal cytology. The clinicopathologic characteristics of the 485 patients, including sex, age, BMI, tumor location, differentiation grade, histological type, lymphatic/venous/neural invasion, level of serum CEA and CA 19-9, and TNM stage were recorded. TNM stage was defined by the AJCC (American Joint Committee on Cancer) TNM staging classification for carcinoma of the stomach (seventh edition, 2010). ECS was defined as the tumor cells infiltrated through the nodal capsule into the perinodal adipose tissue. ECS was detected by hematoxylin-eosin (HE) staining. The histopathology examinations were performed and reviewed by experienced pathologists at the Zhejiang Cancer Hospital.

The study was approved by the Ethics Committee in Research of Zhejiang Cancer Hospital. There was no informed consent because it was retrospective research and would not harm the study’s subjects.

### Follow-up

The patients were followed-up every 3 to 6 months for 1 to 2 years, every 6 to 12 months for 3 to 5 years, and annually thereafter. The duration of follow-up was defined as the interval between the date of the operation and the date of death or the last follow-up. The mean follow-up time was 26.7 months (range 1.3–123.4, SD ± 23.2). Distant metastasis to the parenchymatous organs, such as the liver, lungs, adrenal glands, etc., was based on CT scan or MR. Suspected lesions were ascertained or excluded by biopsy. Metastasis to the bone was based on ECT and/or CT or MR. Abdominal lymph node metastasis was based on a CT scan or MR, but supraclavicular node metastasis was based on fine needle aspiration. Peritoneal or pleural metastasis was mainly based on cytological diagnosis, and some patients were finally diagnosed by surgical exploration for intestinal obstruction.

### Statistical analysis

Statistical analyses were performed using SPSS 17.0 (Statistical Package for the Social Sciences, Chicago, IL, USA). Categorical variables were compared using the chi-squared test or Fisher’s exact test. Continuous variables were analyzed using Mann-Whitney *U* test. Cumulative survival rates were calculated by the Kaplan-Meier method using the log-rank test for comparison. A multivariate Cox regression analysis was carried out to identify independent prognostic factors. Only significant factors from a univariate analysis were used in this multivariate analysis. *P* values less than 0.05 (2-sided) were considered statistically significant.
